# Supplementary material for: Protective Effect of Antrodia cinnamomea Extract against Irradiation-Induced Acute Hepatitis
Source: Int J Mol Sci. 2019 Feb 15;20(4):846. doi: 10.3390/ijms20040846 (PMC6412687; doi:10.3390/ijms20040846)
Supplement: Supplementary file 1 [file ijms-20-00846-s001.pdf]

Supplemental Data

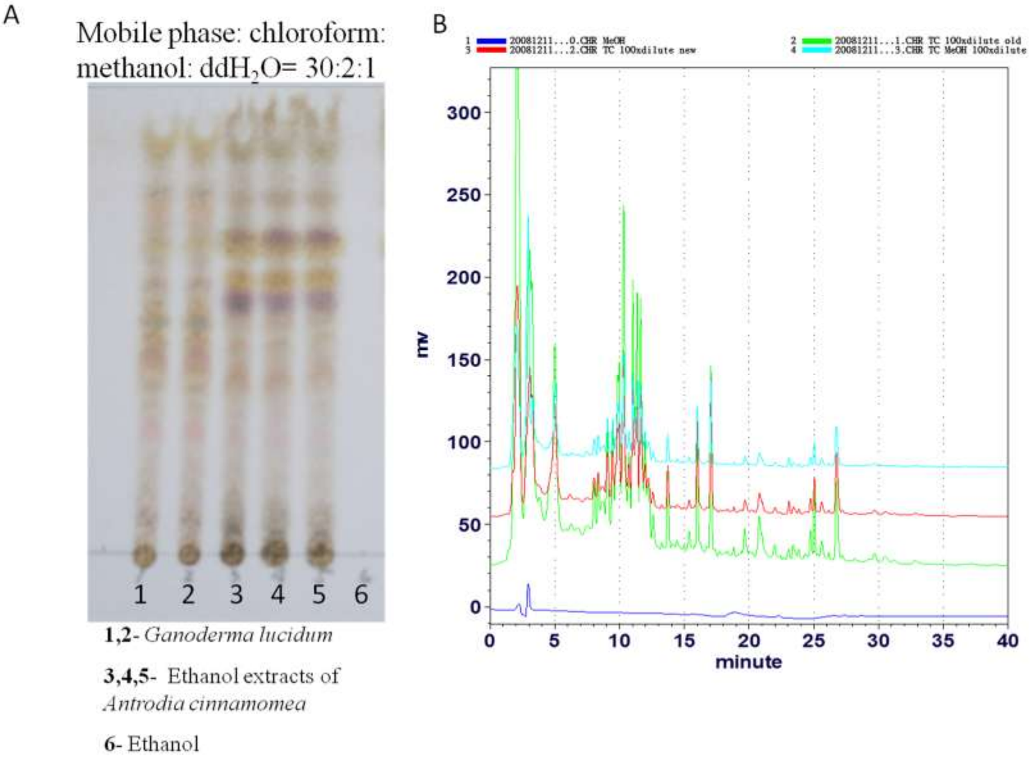

Supplementary Figure S1. **Qualitative analysis of the ethanol extracts of *Antrodia cinnamomea* (ACE).** (A) Thin layer chromatography analysis. ACE was separated in lanes 3, 4, and 5. Extract of *Ganoderma lucidum* was separated in lanes 1 and 2 for comparison. (B) HPLC analysis using ODS-C-18 column. Mobile phase was 30% acetonitrile, 1% acetic acid, 69% ddH₂O. The analysis was performed three times for consistency.

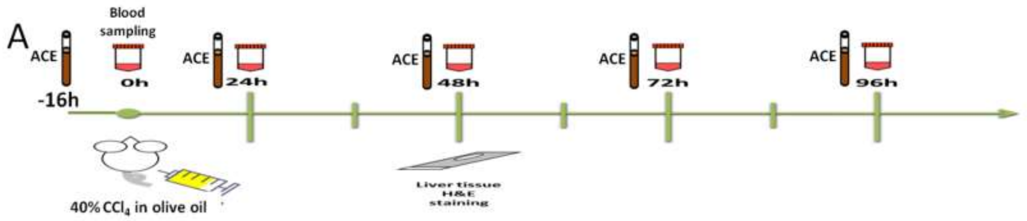

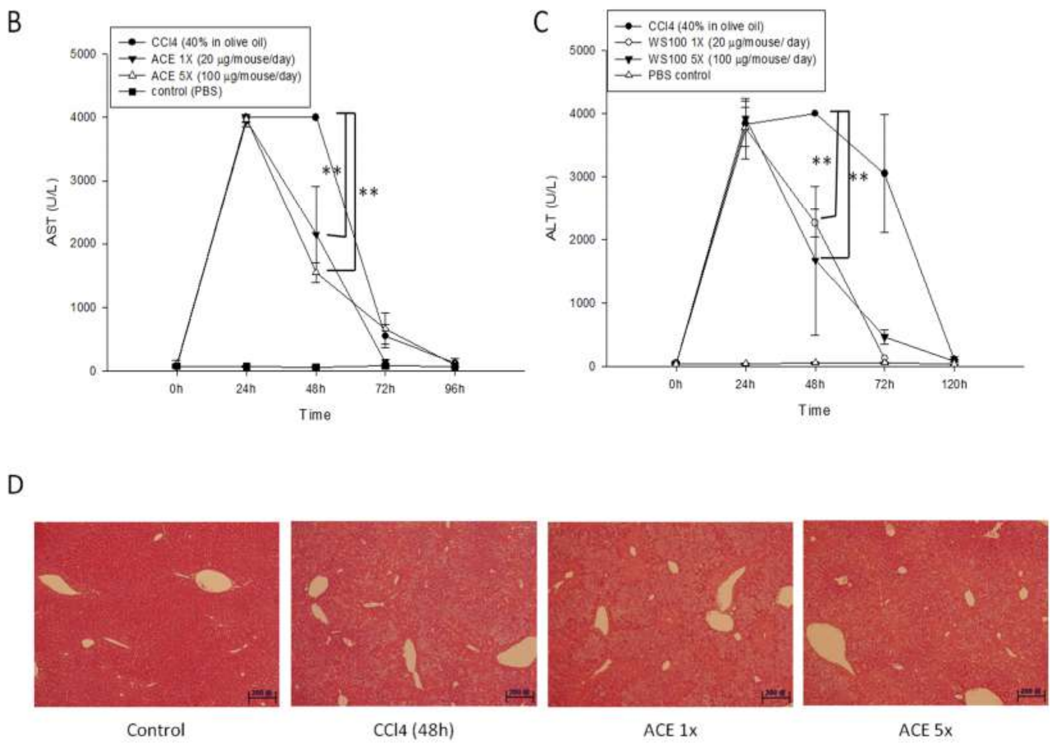

Supplementary Figure S2. **The protective effect of ACE against the CCl_4-induced acute hepatitis.** (A) The scheme of the mouse model study. Acute hepatitis was induced by IP injection of 8-week-old mice with CCl_4 1000 ml/kg (40% dilution in olive oil). ACE was administered by oral gavage at two dosages of 20 and 100 µg/mouse/day. Blood was collected by orbital sinus sampling every 24 h. The first blood sample was collected immediately before induction with CCl_4. Time-course serum levels of the hepatitis markers AST (B) and ALT (C) are shown. The induction of hepatitis and its alleviation by ACE were demonstrated by H&E staining of the mouse liver tissues harvested at 48 h after CCl_4 treatment (D). *, $p < 0.005$; **, $p < 0.001$, as compared with the control group.
